# Comorbidity Between Non-suicidal Self-Injury Disorder and Borderline Personality Disorder in Adolescents: A Graphical Network Approach

**DOI:** 10.3389/fpsyt.2020.580922

**Published:** 2020-11-27

**Authors:** Tinne Buelens, Giulio Costantini, Koen Luyckx, Laurence Claes

**Affiliations:** ^1^Research Unit Clinical Psychology, Faculty of Psychology and Educational Sciences, KU Leuven, Leuven, Belgium; ^2^Department of Psychology, University of Milano-Bicocca, Milan, Italy; ^3^School Psychology and Development in Context, Faculty of Psychology and Educational Sciences, KU Leuven, Leuven, Belgium; ^4^Unit for Professional Training and Service in the Behavioural Sciences, University of the Free State, Bloemfontein, South Africa; ^5^Faculty of Medicine and Health Sciences, University of Antwerp, Antwerp, Belgium

**Keywords:** non-suicidal self-injury (NSSI), NSSI disorder, adolescence, comorbidity, DSM-5, borderline personality disorder, network analysis

## Abstract

In 2013, DSM-5 urged for further research on non-suicidal self-injury (NSSI) and defined NSSI disorder (NSSI-D) for the first time separate from borderline personality disorder (BPD). However, research on the comorbidity between NSSI-D and BPD symptoms is still scarce, especially in adolescent populations. The current study selected 347 adolescents who engaged at least once in NSSI (78.4% girls, *M*_age_ = 15.05) and investigated prevalence, comorbidity, gender differences, and bridge symptoms of NSSI-D and BPD. Network analysis allowed us to visualize the comorbidity structure of NSSI-D and BPD on a symptom-level and revealed which bridge symptoms connected both disorders. Our results supported NSSI-D as significantly distinct from, yet closely related to, BPD in adolescents. Even though girls were more likely to meet the NSSI-D criteria, our findings suggested that the manner in which NSSI-D and BPD symptoms were interconnected, did not differ between girls and boys. Furthermore, loneliness, impulsivity, separation anxiety, frequent thinking about NSSI, and negative affect prior to NSSI were detected as prominent bridge symptoms between NSSI-D and BPD. These bridge symptoms could provide useful targets for early intervention in and prevention of the development of comorbidity between NSSI-D and BPD. Although the current study was limited by a small male sample, these findings do provide novel insights in the complex comorbidity between NSSI-D and BPD symptoms in adolescence.

## Introduction

### Non-suicidal Self-Injury (Disorder)

Non-Suicidal Self-Injury (NSSI) is defined as the socially unacceptable, intentional, and direct injury of one's own body tissue without suicidal intent (1). Common methods of NSSI include cutting, burning, or carving one's own skin (2). In community samples, pooled estimates suggest that 17.2% of adolescents, 13.4% of young adults, and 5.5% of adults report a lifetime history of NSSI (3). In clinical samples, lifetime prevalence rises to 60% in adolescence and 65–80% in adulthood (4, 5). The high prevalence rates of NSSI are alarming, as the behavior has been linked to several mental health conditions. For instance, NSSI is associated with depression, anxiety, rumination, feelings of stigma and shame, and low levels of help seeking (6–8). Moreover, 50–75% of those with a history of NSSI make a suicide attempt at some point in their life (9). Research has shown how NSSI can occur with virtually any mental disorder, although comorbidity rates are particularly high for anxiety and mood disorders, post-traumatic stress disorder, substance use disorder, eating disorders, and personality disorders (10–12). The high prevalence rates and significant mental health implications underscore the necessity for an improved understanding of NSSI (13).

The need for further research on NSSI was formally emphasized with the inclusion of NSSI disorder (NSSI-D) as a “condition requiring further research” in Section III of DSM-5 (14). The newly proposed disorder included six provisional diagnostic criteria (14). First, criterion A specifies that NSSI has to occur for at least 5 days in the past 12 months. Second, criterion B states that the individual must engage in NSSI for one or more of these reasons: to relieve negative thoughts or feelings (B1), to resolve interpersonal difficulties (B2), or to induce a positive state (B3). Third, criterion C indicates that NSSI must be preceded by either negative thoughts or feelings (C1a), conflicts with others (C1b), preoccupation with the behavior that is difficult to resist (C2), or recurrent thoughts about the behavior (C3). Finally, socially acceptable behaviors are excluded (criterion D), the behavior must cause significant distress or interference in the individual's daily life (criterion E), and should not occur solely in the context of another mental disorder (criterion F).

Although research on NSSI has mainly focused on adults and college students, adolescents seem to be particularly at-risk for an NSSI-D diagnosis (3). Based on the limited available data, it has been estimated that 5.6–7.6% of adolescents are eligible for an NSSI-D diagnosis in community samples, compared to 0.2–3% of (young) adults (15–17). Moreover, it has been found that 37.7% of community adolescents with a lifetime history of NSSI meet all six NSSI-D criteria (17). In most studies, the diagnosis was more common in girls than in boys (16). These results may be subject to change, as discussion regarding the exact formulation and clinical relevance of some of the diagnostic criteria and NSSI-D as a separate disorder is still ongoing (17–20). For instance, a recent study suggested that the NSSI-D frequency cut-off should be raised from 5 days to at least 10 days in the past year to clinically meaningful (21, 22). However, a review of 16 empirical studies using the current DSM-5 criteria already found preliminary support for a distinct NSSI-D diagnosis, independent of other closely related mental disorders (16). For instance, in one of the reviewed studies, 80% of adolescents who met the current NSSI-D criteria did not meet criteria for Borderline Personality Disorder (BPD), thus indicating that NSSI-D can occur independently of BPD (23, 24). The distinction between NSSI(-D) and BPD is particularly relevant because NSSI has been historically intertwined with BPD as a prototypical symptom of the disorder (11). More specifically, before the release of DSM-5, NSSI was only mentioned in the DSM as a criterion for BPD.

### Borderline Personality Disorder

BPD is a severe mental disorder that is generally typified by four core features: affective instability, identity problems, negative or unstable interpersonal relationships, and impulsivity or recurrent self-harm (14). Individuals diagnosed with BPD tend to experience strong emotions and can be particularly sensitive to rejection (25), they are more likely to suffer from severe psychosocial impairment such as intense conflict and tumultuous relationships (26), and show high mortality rates due to suicide, with up to 10% of BPD patients committing suicide (27). Epidemiological studies have shown that BPD prevalence rates peak in late adolescence and range from 2 to 3.2% in community adolescents (28, 29), 11% in adolescent outpatients, and 33–49% in adolescent inpatients (30–32). In community samples, most studies suggest an equal prevalence in adolescent boys and girls (28, 33). In clinical samples, prevalence rates are typically cited as higher among girls than boys, although it has been argued that this might be an artifact of sampling or diagnostic biases (34, 35). Importantly, adolescents with BPD are more likely than adults to show “acute” BPD symptoms, such as suicidal ideation and recurrent NSSI (36). Around 61% of adolescents with BPD pathology have engaged in NSSI at least once, making “recurrent NSSI and suicidal behavior” the most commonly met diagnostic criterion for BPD in adolescence (34, 37). In this young at-risk age group, the comorbidity between BPD and NSSI is complex (38). For instance, displaying BPD symptoms indicates greater severity of NSSI based on several parameters (36) such as a younger age of NSSI onset (37, 39), more frequent NSSI episodes (40), and a higher likelihood of repetitive NSSI (41). NSSI in adolescence is considered a key precursor for, or even indicator of, BPD, especially when repetitive and long-lasting NSSI is present (42). Severity of NSSI (i.e., earlier age of onset and longer duration of the behavior) is a risk factor for later BPD (43). On the other hand, the majority of adolescents engaging in NSSI do not meet the criteria for BPD (44, 45). To improve our understanding of comorbidity, the field could benefit from adopting a symptom-level approach of the comorbidity between NSSI-D and BPD. This could clarify whether or not NSSI-D and BPD symptoms cluster together and, most importantly, detect which symptoms drive the high co-occurrence between both diagnoses. Network theory offers a compelling new direction because of its clear symptom-level conceptualization of comorbidity and its statistical tools to model and visualize the approach (46).

### Network Theory as an Innovative View on Comorbidity of Mental Disorders

In 2013, Borsboom and Cramer introduced network theory, a conceptual framework asserting that mental disorders are networks of symptoms influencing each other, rather than symptom sets being caused by an underlying disease entity (47). The network theory innovated analysis of comorbidity (48), because it states that a symptom can directly activate one or more symptoms in other disorder's network, which thus links disorders to each other without the assumption of a latent comorbidity factor. The accompanying statistical technique, network analysis, allows researchers to model and visualize these symptom associations to illuminate the nosology and comorbidity of mental disorders (47).

In network analysis, the graphical output represents each symptom by a *node*. Nodes that tend to co-occur in the data are joined together by connecting *edges*, which results in a web-like constellation or *network* (47). If a group of nodes cluster more strongly among each other than with other nodes, that group is defined as a *community* (49, 50). A community structure analysis therefore offers an innovative way of detecting whether or not the symptoms in a network form statistically discernible symptom clusters (i.e., in our study, an NSSI-D community and a BPD community). Interestingly, certain edges can bridge two disorders by running from a node belonging to one theoretically defined cluster (e.g., NSSI-D) to a node belonging to another cluster (e.g., BPD). These between-cluster nodes are aptly referred to as *bridge symptoms* (46). Bridge symptoms are powerful tools in studying comorbidity, as they provide valuable information regarding the spread of activation between disorders. Specifically, the presence of an identified bridge symptom might indicate a heightened risk for the onset of an additional disorder, or, if both disorders are already present, the bridge symptom might play a role in maintaining the spread of activation between them (51). Albeit connections in networks do not necessarily reflect causal structures, edges can be indicative of potential mutual or directed causal relationships (52, 53). If this is the case, “deactivating” a bridge symptom, for instance by intervention or medication, could be regarded as cutting a crucial connection between comorbid disorders. In other words, successfully treating a bridge symptom could result in a decrease in symptom-level associations both within- and between-disorders (46, 51). Up until recently, researchers had to rely on subjective visual inspection of a network to detect bridge symptoms (46). However, in 2019 Jones et al. developed and validated a quantitative index to identify bridge symptoms and to measure their centrality between theoretically defined clusters.

### Research Aims and Hypotheses

Embracing these state-of-the-art techniques, the aim of the present study was fourfold: (1) describe prevalence rates of (the comorbidity between) NSSI-D and BPD symptomatology, (2) investigate whether or not NSSI-D and BPD can be distinguished from one another in a network structure, (3) explore potential gender differences in (the comorbidity of) the NSSI-D and BPD network, and (4) identify specific bridge symptoms through which pathology is most likely to spread between NSSI-D and BPD symptom clusters. First, concerning prevalence rates, we tentatively hypothesized that the percentage of individuals scoring above the BPD cut-off in the current sample (i.e., community adolescents engaging in NSSI) would be between percentages found in community adolescents [i.e., 2–3.2%, (28, 29)] and in adolescent outpatients [i.e., 11%, (30)]. Concerning the second research aim, we hypothesized based on a review of the empirical NSSI-D literature (16) that NSSI-D and BPD symptoms (nodes) would split into at least two statistically discernible communities without symptoms from NSSI-D belonging to the BPD community or vice versa. Regarding the third research aim, we expected more girls than boys to be eligible for an NSSI-D diagnosis (16), but we did not expect gender differences in the percentage of boys and girls scoring above the BPD cut-off in this sample (28, 33). To the best of our knowledge, no research is currently available on gender differences in the comorbidity between BPD and NSSI-D symptomatology. Lastly, as no previous research is currently available on potential bridge symptoms between NSSI-D and BPD, no specific hypotheses could be formulated.

## Materials and Methods

### Procedure

The current study is part of a research project in which eight secondary schools took part, all located in Flanders, Belgium (17). Across all eight schools, we contacted the parents of 3,483 students and distributed informed consent forms among them. A total of 2,313 (66.4%) students received active parental consent and were subsequently invited to partake in the study. The 2,162 (93.5%) students who agreed to participate received an assent form, a questionnaire booklet, and an envelope. The data collection took place during school hours, with the researchers present at all time. After signing the assent form and filling out all questionnaires, the students returned these documents in a sealed envelope to the researchers. Students who were absent on the day of assessment were contacted by e-mail to complete an online version of the study. All participants received a movie ticket as compensation, as well as a letter with contact details of the school counselor and several mental health services. The study was approved by the Ethics Committee at the University of Leuven.

### Participants

Out of the 2,162 participating students, we selected only those who reported having ever engaged in NSSI (i.e., “I have at least once engaged in self-injury without the intent to die”) and who completed the BPD questionnaire. This resulted in a final sample of 347 students (78.4% female) between the ages of 12 and 20 (*M* = 15.05, *SD* = 1.83). The vast majority of students identified as Belgian (93.1%). About half of the students lived with both parents (53.0%, *n* = 184), the remaining students had divorced parents and/or lived in a blended family (40.4%, *n* = 132) or indicated to have another home environment (9%, *n* = 31).

### Measures

#### Non-suicidal Self-Injury Disorder

Lifetime NSSI was assessed using the single-item screening measure “Have you ever engaged in self-injury without an intent to die?” Those who answered affirmatively responded to follow-up questions regarding frequency and recency of NSSI, current NSSI, age of NSSI onset, and different NSSI behaviors (i.e., scratching, carving, cutting, burning, rubbing the skin, self-hitting, pricking/piercing the skin, and banging the head). Additionally, a set of questions assessing DSM-5 criteria for NSSI-D was included. We used questions that explicitly assessed all NSSI-D criteria (A, B, C, D, E, and F), with the wording of these items matching the DSM-5 criteria as closely as possible [see Buelens et al. (17) for an overview of the exact questions]. Furthermore, since previous research indicated that criterion C1 contains two elements that are considerably different from each other (17), we additionally split criterion C1 into C1*a* (negative feelings or thoughts) and C1*b* (conflicts with others) to assess this symptom more accurately. We used a dichotomous approach when describing prevalence rates [i.e., fulfilling (1) or not fulfilling (0) the criterion], while we used the continuous scores on each criterion in the network analyses. For all DSM-5 criteria together, a KR-20 reliability coefficient of 0.667 was found, which is close to the 0.7 cut-off for acceptable internal consistency (54).

#### Borderline Personality Disorder Symptomatology

The brief Borderline Personality Features Scale for Children [BPFSC-11; (55)] was used to assess BPD symptomatology. The questionnaire consists of 11 items scored on a 5-point Likert scale ranging from 0 (*never true for me*) to 4 *(always true for me*) and results in a unidimensional sum score ranging from 0 to 44 or mean score ranging from 0 to 4 (56). A higher mean score indicates more BPD symptomatology. The BPFSC-11 had a Cronbach's alpha of 0.79 in the current study, which is comparable to previous research (56). Next to the continuous mean score, we created a dichotomous cut-off score (1 = above the BPD cut-off, 0 = below the BPD cut-off) as recommended by previous sensitivity and specificity analyses on the BPFSC-11, which indicated the ideal cut-off value to be 34 out of the maximum sum score of 44 (55). We used the dichotomous score when describing percentages of adolescents scoring above and below the cut-off, while we used the continuous BPD score in the network analyses. The BPFSC-11 does not include items assessing NSSI.

### Statistical Analyses

To address the first research aim, we used SPSS version 26 (57) to conduct descriptive analyses and compute prevalence rates of (the comorbidity between) NSSI-D and BPD symptoms. Research aims two to four were addressed using R (58) to conduct network analyses. For these analyses, participants who had six or more missing values were removed (*n* = 10) and the remaining 22 missing values out of the 7,751 datapoints were imputed using the *mice* R package (59).

As the second research aim was to investigate whether or not NSSI-D and BPD would occur as statistically discernible clusters of symptoms (nodes), we modeled a weighted, undirected graphical LASSO network using *qgraph* (60). We used the Extended Bayesian Information Criterion (EBIC), with the γ hyperparameter at 0.25, to set the amount of LASSO regularization (61). We then conducted a community structure analysis using the Walktrap algorithm, as implemented in the *igraph* R package (62, 63). Expected influence (EI) was used as a centrality measure, as it accounts for the presence of potential negative edges in the network by not taking the absolute value of edges before summing them (46, 64). The robustness (accuracy and stability) was tested by the bootstrapping procedure in the *bootnet* R package (65, 66). This procedure estimates a 95% confidence interval around the edges to estimate accuracy and provides a correlation-stability (CS) coefficient to assess whether or not the centrality indices (e.g., EI) are stable enough to be interpreted (65, 66). Namely, the CS-coefficient represents the proportion of participants that can be removed from the sample in case-dropping bootstrap resamples, such that the resulting EI indices have a 95% probability to correlate ≥ 0.7 with the original EI index (65). As a rule of thumb, a CS-coefficient below 0.25 indicates insufficient stability and warns against interpreting the centrality indices. A CS-coefficient above 0.50 indicates good stability (66).

To address the third research aim concerning potential gender differences in the network, we used the Network Comparison Test (NCT, γ = 0.25) from the *NetworkComparisonTest* R package (67) to investigate if the network structure and global strength were significantly different between boys and girls in the sample. The NCT allows us to assess the difference between the male and the female network based on network invariance, global strength invariance, edge invariance and centrality invariance.

Finally, to reach the fourth research aim, we used the *networktools* R package (68) to detect bridge symptoms between NSSI-D and BPD. As we were interested in the comorbidity between these two disorders, we specified to the network model which symptoms belonged to NSSI-D and which symptoms belonged to BPD. We then used bridge EI as a centrality measure to indicate which symptoms operate as bridges between the two theoretically defined symptom sets (64). We computed the CS-coefficient for bridge EI using the same case-dropping bootstrap resample as described above.

## Results

### Descriptive Statistics

#### Non-suicidal Self-Injury

At the moment of assessment, 4.6% (*n* = 16) of participants reported having engaged in NSSI that same day and/or the day before, 8.9% (*n* = 31) reported having engaged in NSSI a couple of days ago, 11.8% (*n* = 41) a week ago, 11.5% (*n* = 40) a month ago, 35.2% (*n* = 122) several months ago, and 27.1% (*n* = 94) reported having engaged in NSSI over a year ago. Three participants did not answer this question. A total of 20.7% (*n* = 72) of the participants described themselves as “currently engaging in NSSI.” The most common methods of NSSI were cutting and carving one's own skin, with 53.0% (*n* = 184) and 51.0% (*n* = 177) of the sample indicating that they engaged in these behaviors at least once. The other behaviors were hitting (30.8%, *n* = 107), scratching (26.5%, *n* = 92), head banging (25.4%, *n* = 88), pricking/piercing (23.1%, *n* = 80), rubbing (11.8%, *n* = 41), and burning the skin (8.4%, *n* = 29). The mean age of NSSI onset was 12.87 years (*SD* = 2.03), which did not significantly differ between boys (*M*_age_ = 12.76, *SD* = 2.23) and girls [*M*_age_ = 12.90, *SD* = 1.98; *F*_(1, 332)_ = 1.456, *p* = 0.619].

#### Non-suicidal Self-Injury Disorder

Buelens et al. (17) provides more details on the diagnostic NSSI-D criteria in this sample. In short, a total of 37.8% (*n* = 131) of the participants adhered to all DSM-5 criteria for NSSI-D, whereas 59.9% (*n* = 208) was at least one criterion short of being eligible for an NSSI-D diagnosis. 2.3% (*n* = 8) of participants could not be classified on absence of presence of NSSI-D due to missing data on the NSSI-D criteria. When considered dichotomously, criterion A was met by 51.6% of the sample, criterion B by 86.9%, criterion C by 97.9%, criterion D by 100%, criterion E by 78.6%, and criterion F by 99.1% of the sample. Significantly more girls were eligible for an NSSI-D diagnosis (*n* = 111 out of 265 girls, 41.89%) compared boys (*n* = 20 out of 74 boys, 27.03%) according to the assessed DSM-5 criteria [*X*^2^(1) = 5.39, *p* = 0.020].

#### Borderline Personality Disorder

The mean score for BPD symptomatology was 2.16 (*SD* = 0.66) and was significantly higher for girls (*M* = 2.22, *SD* = 0.64) than boys [*M* = 1.95, *SD* =0.70; *F*_(1, 344)_ = 1.30, *p* = 0.002]. There was no significant effect of age [*F*_(8, 337)_ = 0.709, *p* = 0.684] on the mean score for BPD symptomatology. We additionally performed analyses using the dichotomous cut-off variable (1 = above the BPD cut-off, 0 = below the BPD cut-off). A total of 6.6% (*n* = 23) of the sample scored above the BPD cut-off, 93.1% (*n* = 323) scored below the cut-off, and 0.3% (*n* = 1) could not be classified due to missing data. Although a higher percentage of girls (*n* = 21 out of 271 girls, 7.75%) scored above the BPD cut-off compared to boys (*n* = 2 out of 75 boys, 2.67%), this difference did not reach statistical significance [*X*^2^ (1) = 2.45, *p* = 0.118].

#### Comorbidity

In the cross tabulation of NSSI-D and BPD (Table 1), all adjusted standardized residuals exceeded |2|, indicating significant discrepancies between the observed and expected frequencies. Out of the 23 participants who scored above the BPD cut-off, 60.87% (*n* = 14) met the NSSI-D diagnosis as well, the remaining 39.13% (*n* = 9) did not meet the criteria for an NSSI-D diagnosis. Out of the 131 participants who met the NSSI-D diagnosis, 10.68% (*n* = 14) scored above the BPD cut-off as well, while 89.31% (*n* = 117) scored below the BPD cut-off. These differences were statistically significant [*X*^2^ (1) = 5.08, *p* = 0.024], with a higher probability to observe NSSI-D in the BPD group.

**Table 1:** Cross tabulation of NSSI-D and BPD.

	**BPD**	**No BPD**	**Total**
NSSI-D	**14** (2.3)	**117** (−2.3)	131
No NSSI-D	**9** (−2.3)	**198** (2.3)	207
Total	23	315	

*NSSI-D, Non-suicidal self-injury disorder; BPD, Borderline personality disorder*.

*Adjusted standardized residuals are in parentheses*.

*Number of participants in this category are in bold*.

Table 2 shows the Pearson correlations between all NSSI-D criteria and all BPD symptoms.

**Table 2:** Correlation coefficients between all study variables.

	**Strong**	**Back**	**Miss**	**Leave**	**Change**	**Lonely**	**Hurt**	**Letdown**	**Mean**	**Careless**	**Nothink**
A	0.136^*^	0.219^**^	0.230^**^	0.188^**^	0.134^*^	0.261^**^	0.172^**^	0.148^**^	0.007	0.046	0.105
B1	0.077	0.198^**^	0.186^**^	0.228^**^	0.166^**^	0.259^**^	0.157^**^	0.184^**^	0.071	0.119^*^	0.043
B2	0.037	0.118^*^	0.058	0.123^*^	0.07	0.095	0.014	0.01	0.042	0.026	0.073
B3	0.105	0.074	0.151^**^	0.086	0.065	0.065	0.103	0.072	0.074	0.005	0.123^*^
C1a	0.185^**^	0.308^**^	0.252^**^	0.276^**^	0.163^**^	0.291^**^	0.210^**^	0.258^**^	0.117^*^	0.173^**^	0.164^**^
C1b	0.213^**^	0.181^**^	0.108^*^	0.159^**^	0.121^*^	0.099	0.168^**^	0.194^**^	0.037	0.109^*^	0.178^**^
C2	0.231^**^	0.265^**^	0.245^**^	0.189^**^	0.206^**^	0.216^**^	0.231^**^	0.151^**^	0.079	0.163^**^	0.165^**^
C3	0.128^*^	0.321^**^	0.344^**^	0.246^**^	0.146^**^	0.389^**^	0.172^**^	0.192^**^	−0.005	0.174^**^	0.110^*^
E1	0.136^*^	0.096	0.184^**^	0.151^**^	0.073	0.116^*^	0.119^*^	0.121^*^	0.052	0.129^*^	0.152^**^
E2	0.155^**^	0.169^**^	0.159^**^	0.220^**^	0.077	0.193^**^	0.141^**^	0.130^*^	0.105	0.163^**^	0.251^**^
E3	0.125^*^	0.119^*^	0.152^**^	0.147^**^	0.071	0.135^*^	0.132^*^	0.128^*^	0.099	0.061	0.195^**^
E4	0.164^**^	0.058	0.126^*^	0.153^**^	0.043	0.091	0.091	0.114^*^	0.05	0.063	0.049

* *p < 0.05*.

** *p < 0.01*.

**Figure 1:**
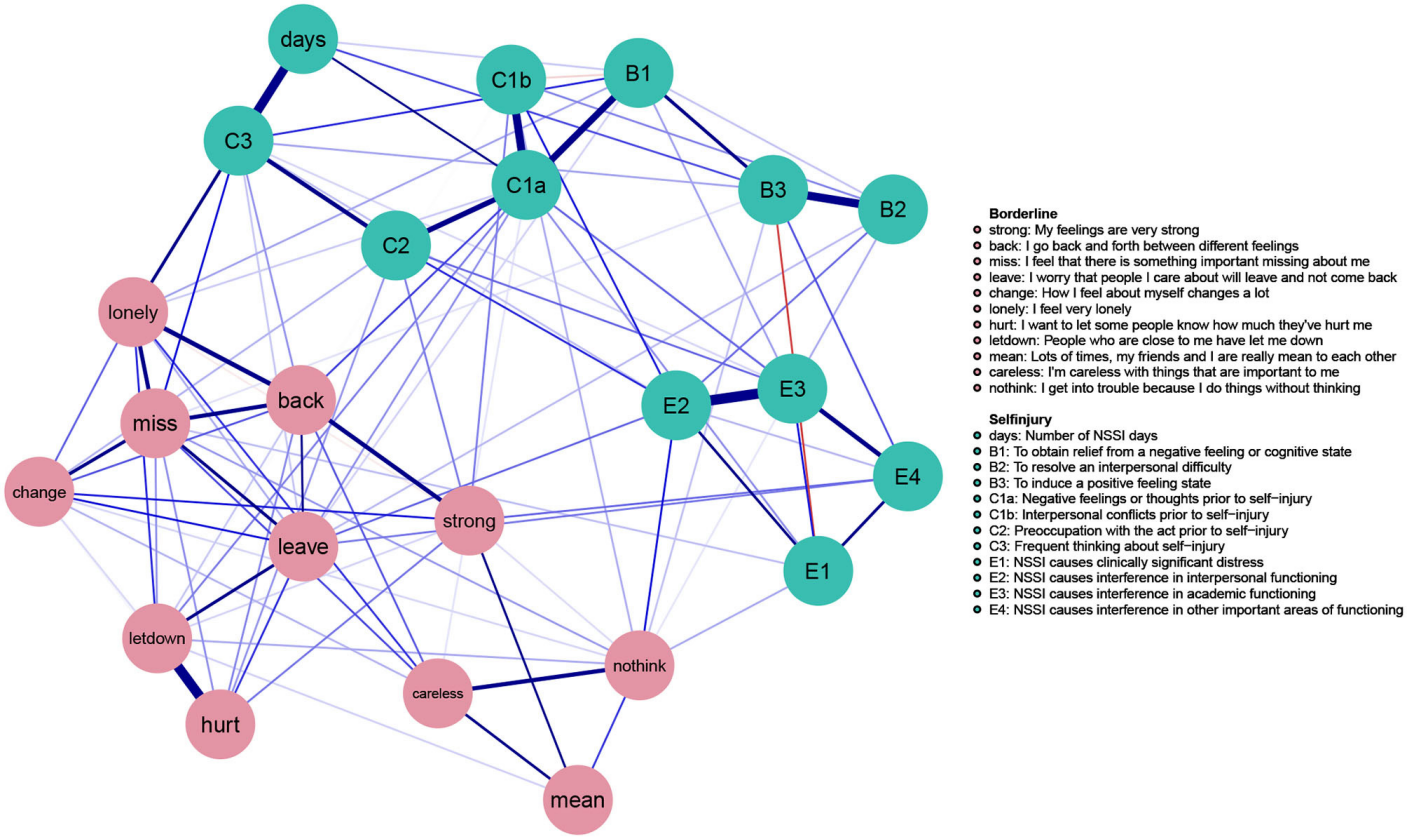
Full gLASSO network.

*Significant correlations are marked in bold. For the full legend, see Figure 1*.

### Graphical Network Analysis

Figure 1 visualizes the EBIC gLASSO network (γ =0.25) based on the 12 NSSI-D items (in green) and the 11 BPD items (in pink). Positive regularized edges are depicted in blue, negative regularized edges are in red. The exact values of each edge, as well as the bootstrapped confidence intervals are reported in Supplementary Table 1. All 99 edges in this network were positive, with the exception of a small negative edge (−0.09) between B3 (*I engage in NSSI to induce a positive feeling state*) and E1 (*NSSI causes clinically significant distress*), a small negative edge (−0.02) between C1b (*I engage in NSSI to obtain relief from a negative feeling of cognitive state*) and B1 (*interpersonal conflict takes place prior to NSSI*), as well as a small edge (−0.01) between nodes *lonely* and *strong*. Regarding the stability of the centrality measures, the CS-coefficient as calculated by the case-dropping bootstrap resample was 0.65 for EI and 0.401 for bridge EI. Thus, the CS-coefficient for EI stayed above the desired 0.50 threshold and the coefficient for bridge EI remained well-above the lower limit of 0.25, making it justifiable to interpret EI results for this network (66), albeit with some caution in the case of bridge EI. Regarding overall EI, nodes C1a (n*egative feelings prior to NSSI)* C3 (*frequent thinking about NSSI)*, E2 (*NSSI causes interference in interpersonal functioning)*, back (*I go back and forth between different feelings)*, and miss (*I feel that something important is missing about me*) had the highest expected influence in the full network (see the second panel of Figure 3). These five symptoms thus had strong and numerous connections to other symptoms and acted as hubs connecting otherwise disparate symptoms to one another (46). The lowest EI was found for mean (*lots of times, my friends and I are really mean to each other)* and E1 (*NSSI causes clinically significant distress*), indicating that both symptoms operated in the periphery of the network, with few and/or weak connections to other symptoms (46).

#### Community Structure Analysis

Upon visual inspection (Figure 1), the NSSI-D items clearly clustered together at the top half of the network while the BPD items clustered together at the lower half of the network. Even though both sets of symptoms were substantially interrelated with each other, no NSSI-D symptoms were nested within the group of BPD symptoms or vice versa.

**Figure 2:**
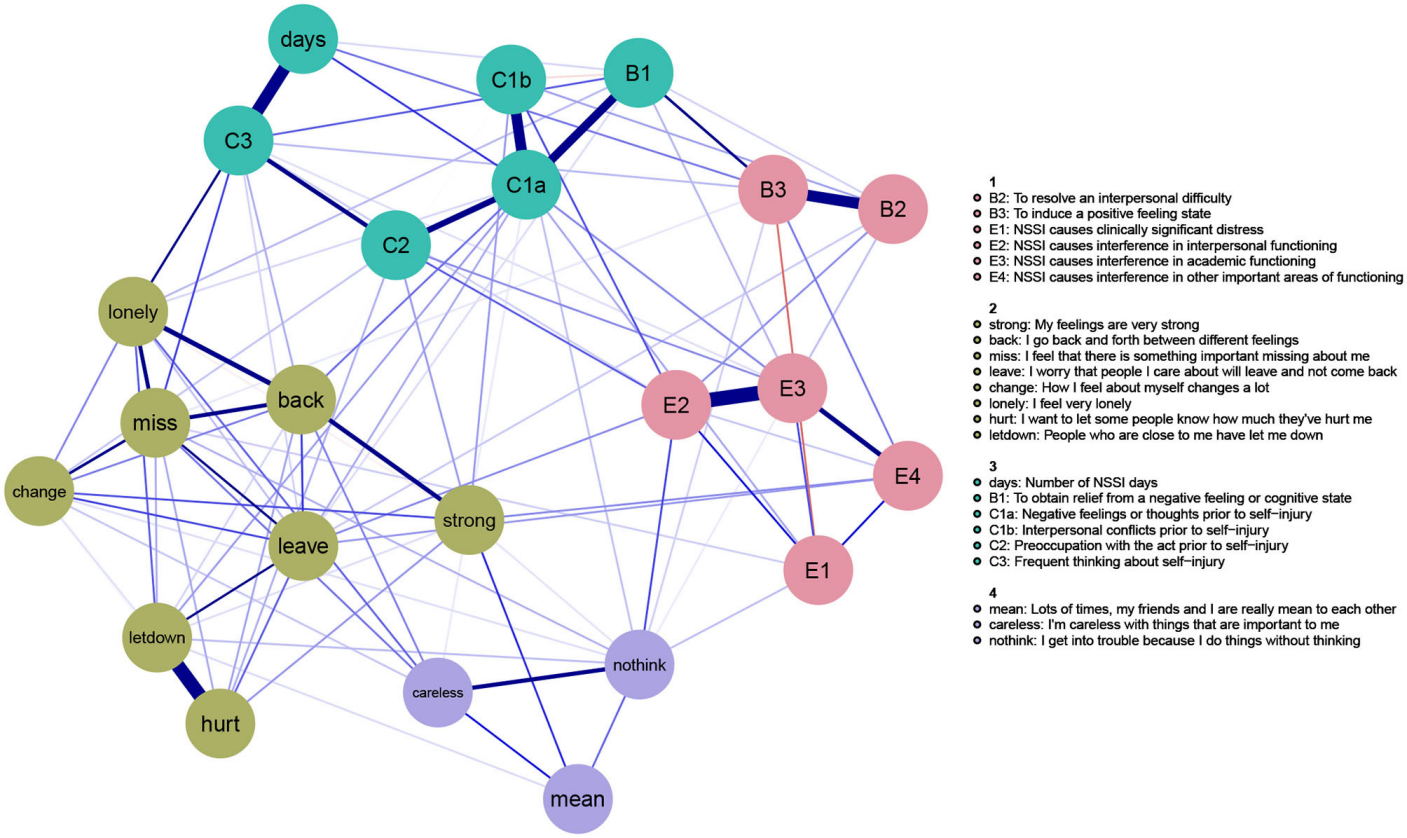
Community structure gLASSO network.

The results of the community structure analysis (Figure 2) corroborated this visual interpretation of the network. Namely, our results showed two communities consisting exclusively of NSSI-D symptoms and two communities consisting exclusively of BPD symptoms, without any overlap (i.e., no NSSI-D symptoms were part of a BPD community or vice versa). For NSSI-D, the E-criteria [*NSSI causes clinical* (E1), *interpersonal* (E2), *academic* (E3), *other* (E4) *distress*], and two of the B-criteria [*engaging in NSSI to resolve interpersonal difficulties* (B2) or *to induce a positive state* (B3)] formed one community (see Figure 2, depicted in pink). The remaining criteria [*engaging in NSSI to relieve negative feelings/thoughts* (B1); *number of NSSI days* (A, days); *negative feelings* (C1a), *conflicts* (C1b), *preoccupation with NSSI* (C2), and *frequent thinking about NSSI* (C3)] constituted the third NSSI-D community (depicted in blue). Regarding BPD, the impulsivity symptoms (*I'm careless with things that are important to me* (Careless) and *I get into trouble because I do things without thinking* (nothink) grouped together with *my friends and I are really mean to each other* (mean) into the first BPD community (depicted in purple). The second BPD community (depicted in green) consisted of the remaining BPD symptoms.

#### Gender Differences in the Network

The network invariance test indicated no significant differences in network structure (*M* = 0.46, *p* = 0.215) and no significant differences between girls and boys in global strength across networks (girls: 9.23, boys: 0, *s* = 9.23, *p* = 0.243). However, these results should be interpreted cautiously, as the lack of a significant gender differences might be a result of low power due to the small number of boys in our sample (21.6%, *n* = 75).

#### Bridge Symptoms

**Figure 3:**
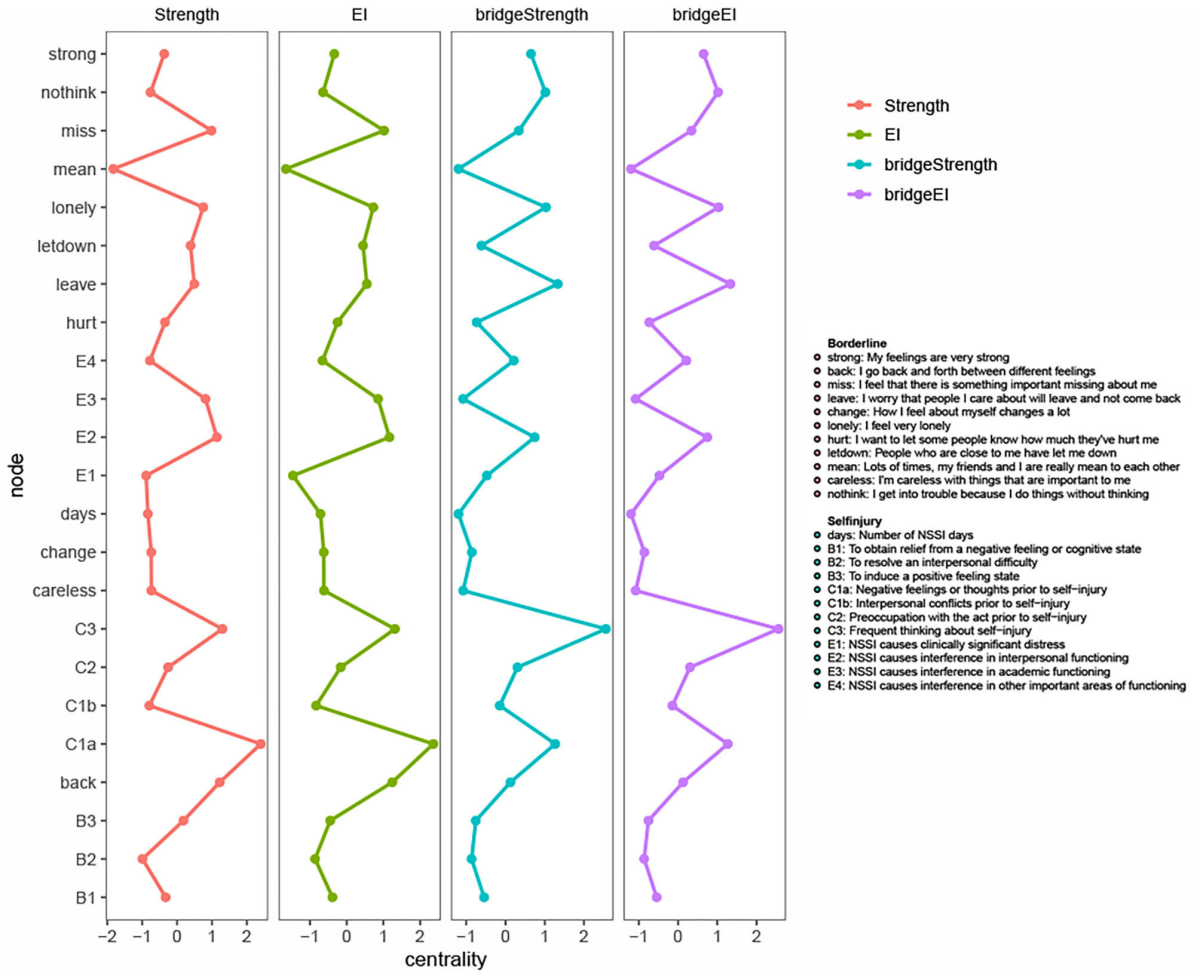
(Bridge) strength and (bridge) expected influence. Centrality measures are visualized using standardized values to facilitate comparison. The x-axis represents standardized centrality values, the y-axis represents each node. The full definition of each node can be found in the legend. *EI*, expected influence.

Figure 3 summarizes the standardized centrality measures for each of the 23 symptoms included in the network. For the sake of completeness, we included both strength centrality and expected influence (EI). However, because of the small proportion of negative edges in the network, strength centrality and EI were nearly identical for the overall measures (*r*_strngxEI_ = 0.98) and exactly identical for the bridge measures (*r*_strngxEI_ = 1), because all negative edges connected nodes within the same cluster. Importantly however, as the CS-coefficient for bridge EI was below the 0.50 threshold, the results below should be interpreted with some caution.

The highest bridge EI was found for C1a (*negative feelings or thoughts prior to NSSI*), C3 (*frequent thinking about NSSI*), leave (*I worry that people I care about will leave and not come back*), lonely (*I feel very lonely*), and nothink (*I get into trouble because I do things without thinking*). This could indicate that these five nodes might have many and/or strong inter-cluster edges bridging the theoretically defined clusters of NSSI-D and BPD symptoms. The lowest bridge EI was found for days (*number of NSSI days*), E4 (*NSSI causes interference in other important areas of functioning)*, mean (*my friends and I are really mean to each other*) and careless (*I'm careless with things that are important to me*). This might indicate that these four nodes did not play a significant role in connecting NSSI-D and BPD symptoms. This could either be due to overall low EI (as is the case for *mean*), or to being mainly connected to nodes within the same cluster (as is the case for *careless* in the BPD cluster and *E4* and *days* in the NSSI-D cluster). The latter could indicate that these symptoms are potentially less relevant in the comorbidity between NSSI-D and BPD, even though they could play a considerable role within each disorder.

## Discussion

In 2013, DSM-5 urged for further research on NSSI-D and represented NSSI for the first time distinct from BPD (14). However, research on the comorbidity between NSSI-D and BPD symptoms is still scarce, especially in adolescent populations, where the symptoms of both disorders tend to be more acute and more prevalent than in adulthood (36). Therefore, the current study selected 347 adolescents who engaged at least once in NSSI to address four research aims regarding prevalence, comorbidity, gender differences, and bridge symptoms of NSSI-D and BPD.

First, our results showed that 6.6% in this specific sample (i.e., community adolescents with a history of NSSI) scored above the BPD cut-off, which turned out to be higher than the 2–3% previously found in community adolescents (28, 29), but lower than the 11% previously found in outpatient adolescents (30). Regarding the co-occurrence of BPD with NSSI-D, our results showed that 60.87% of adolescents who scored above the BPD cut-off were eligible for an NSSI-D diagnosis as well. To the best of our knowledge, the current study is the first to report the co-occurrence of BPD with NSSI-D in community adolescents. Previous research, however, did already indicate that out of those adolescents who presented with BPD symptomatology, 61% had at least once engaged in NSSI (34, 37). Considering the reverse direction (i.e., the co-occurrence of NSSI-D with BPD), our results showed that 37.14% of adolescents eligible for NSSI-D scored above the BPD cut-off as well. This percentage is just below the 44.4% reported recently by Zetterqvist et al. (69). The slight difference might be due to the fact that our study investigated community adolescents, whereas Zetterqvist et al. studied adolescent outpatients.

Second, to investigate comorbidity in more detail, we modeled the symptoms of NSSI-D and BPD together in one network of inter-symptom relations. The network showed how NSSI-D and BPD symptoms were closely interrelated, with a total of 98 connections running to and from the 23 symptoms included in the network. Despite this interconnectedness, a community structure analysis revealed that NSSI-D and BPD symptoms reliably split into separate communities, where no symptoms from NSSI-D ended up in the BPD community nor vice versa. These results confirm earlier research, which found NSSI-D to occur both together with and independently of BPD (16, 69). As a previous study showed that the overlap between BPD and NSSI-D is similar to the overlap between BPD and other disorders (24), these findings seem to strengthen the validity of distinct, yet related diagnoses (24, 69). Two additional findings emerged from the community structure analysis regarding the clustering of symptoms within NSSI-D. First, criterion B1 (*engaging in NSSI to relieve negative feelings/thoughts*) did not group together with the other B-criteria, but rather formed a community with the A-criterion (*number of NSSI days*) and C-criteria [i.e., *negative feelings* (C1a), *conflicts* (C1b), *preoccupation with NSSI* (C2), and *frequent thinking about NSSI* (C3)]. This could be due to the particularly strong edge connecting *engaging in NSSI to relieve negative feelings/thoughts* (B1) and *experiencing negative thoughts/feelings prior to NSSI* (C1a), which reflects previous research with this sample reporting an almost complete overlap of B1 with C1a (17). Second, criterion A (*the number of days one engaged in NSSI in the last year*) showed relatively low EI and very low bridge EI. This is likely due to the strong connection of criterion A with C3 (*frequent thinking about NSSI, even when it is not acted upon*): the variance in the number of days seems to be explained to a large extent by the thoughts one has regarding NSSI.

Third, we investigated potential gender differences in (the comorbidity of) NSSI-D and BPD symptoms. Confirming our hypotheses based on previous literature (16, 28, 33), the current study found significantly more girls than boys being eligible for an NSSI-D diagnosis, but no significant gender difference in the BPD cut-off. Moreover, our results showed no significant gender differences in the network of NSSI-D and BPD symptoms. This could indicate that the overall comorbidity structure of NSSI-D and BPD, as well as the strength of the connections between the symptoms, remains alike for boys and girls in a community sample. Thus, even though girls are more likely to meet the NSSI-D criteria, our results tentatively suggest that the manner in which NSSI-D and BPD symptoms are interconnected does not differ between girls and boys. Importantly however, the lack of a significant gender differences in the current study could also be ascribed to the particularly small number of males in our sample.

Fourth, the current study identified the five bridge symptoms through which pathology was most likely to spread to or from NSSI-D and BPD symptoms: negative feelings/thoughts prior to NSSI (C1a), frequent thinking about NSSI (C3), separation anxiety (leave), loneliness (lonely), and impulsivity (nothink). The identification of bridge symptoms can clarify why comorbidities occur in some adolescents, but not in others (70). For instance, our results showed *I feel very lonely* (lonely) to be one of the five main bridge symptoms connecting BPD to NSSI-D symptoms (i.e., high bridge EI). If future research could replicate this finding, it could indicate that an adolescent who feels very lonely would be at greater risk for NSSI-D compared to an adolescent with equally severe BPD features, but who does not feel particularly lonely (70). Loneliness standing out as a potential bridge between BPD and NSSI-D symptoms is supported by earlier work, which reported elevated loneliness in NSSI (71, 72) and BPD (73–75), potentially due to the association of loneliness with depression as a comorbid diagnosis for NSSI and BPD (76). Moreover, previous studies suggested that being alone increases self-reflection (77) which, for at-risk adolescents, could trigger an emotional cascade of rumination, depressive feelings, and potentially NSSI (6).

Relatedly, our results showed that the BPD symptom *I feel very lonely* had its strongest connection to NSSI-D with *frequent thinking about NSSI* (C3), which in itself showed up as one of the five strongest bridge symptoms (i.e., high bridge EI). Thus, *frequent thinking about NSSI* (C3) potentially operates as an important gateway from NSSI-D to BPD. In addition, our results revealed how this symptom was highly influential -and sufficiently stable- in the overall network (i.e., high EI). In other words, *frequent thinking about NSSI* (C3) additionally acted as a central hub in the overall network, with strong and numerous connections to symptoms of both NSSI-D and BPD. Noteworthy, previous research has shown that teaching coping skills to reduce and resist frequent NSSI thoughts and urges is a key component of successful treatment for NSSI (78, 79).

Similarly, *experiencing negative thoughts or feelings prior to engaging in NSSI* (C1a) showed up as one of the five main bridge symptoms (i.e., high bridge EI) as well as one of the most influential symptoms in the overall network (i.e., high EI). This neatly aligns with previous research indicating that, on the one hand, engaging in NSSI to relieve negative thoughts and feelings is the most commonly reported function of NSSI (80, 81) and, on the other hand, adolescents with BPD features tend to report particularly strong negative emotions (25).

Finally, BPD symptoms *I worry that people will leave and not come back* (leave) and *I get into trouble because I do things without thinking* (nothink) showed a different pattern: unlike C1a and C3 these symptoms did not stand out in the overall network (i.e., they showed moderate EI), but they did come up as the final two main bridge symptoms (i.e., high bridge EI) connecting BPD to NSSI-D. These bridge symptoms, BPD symptoms referring to separation anxiety (leave) and impulsivity (nothink), thus could indicate that very anxious or very impulsive adolescents would be at greater risk for NSSI-D, compared to adolescents with equally severe BPD features, but who show less separation anxiety or are less impulsive. This finding extents previous research reporting that separation from parents before the age of 15 increases risk for NSSI and that, among all BPD features, impulsivity showed the strongest association with NSSI frequency (82).

The current study adhered to several recommendations stemming from the extensive discussion on network replicability (83, 84). We provided robustness checks (accuracy and stability) with a bootstrapping procedure and, where necessary, warranted against overinterpreting results with insufficient stability. Despite these precautions, our research was not without limitations. First, our sample size, relatively small considering the high statistical power necessary for these analyses, could have led to increased instability in the LASSO network. Particularly the small number of males in our sample, a common issue when researching both NSSI-D and BPD (34, 35), is likely to be the underlying reason for the insignificant gender differences in the network. Future research with larger sample sizes and more equal numbers of boys and girls should aim to replicate this analysis. Second, NSSI-D showed low internal consistency and measuring NSSI-D and BPD solely with self-report questionnaires is limited and could result in reporting bias (85). However, NSSI is typically secretive (86) and parents or teachers are often unaware of the adolescent's engagement in the behavior (87), which makes NSSI(-D) difficult to assess by other informants. Future research could embrace a multi-method approach and include diagnostic clinical interviews to allow for differential diagnostics and/or behavioral measures to assess NSSI-D and BPD more accurately. Third, our results might not be generalizable to clinical, particularly inpatient, samples. Fourth, both NSSI(-D) and BPD symptoms show high comorbidity with other diagnoses, such as major depressive disorder, substance use disorders, anxiety disorders, and eating disorders (76). Future research could aim to replicate our findings while additionally controlling for other diagnostic comorbidity. Finally, the cross-sectional nature of our data limits the conclusions that can be drawn. Future longitudinal research will allow us to make stronger assumptions regarding long-term symptom interactions and, noteworthy, directionality and causality. Namely, by using time-series data on a group level or on an individual level, specific nodes could be targeted by experimental manipulations to test for causality in the network (70).

## Data Availability Statement

The raw data supporting the conclusions of this article will be made available by the authors, without undue reservation.

## Ethics Statement

The studies involving human participants were reviewed and approved by Ethics Committee at the University of Leuven (G-2017 01 755). Written informed consent to participate in this study was provided by the participants' legal guardian/next of kin.

## Author Contributions

TB was involved in the conception, design of the study, the acquisition, initial analysis, interpretation of the data, the drafting, and revisions of the manuscript. GC made substantial contributions to the analysis, interpretation of the data, and was involved in the revisions of the manuscript. KL and LC were involved in the funding acquisition, conception and design of the study, and revisions of the manuscript. All authors read and approved the final manuscript.

## Conflict of Interest

The authors declare that the research was conducted in the absence of any commercial or financial relationships that could be construed as a potential conflict of interest.
