# Supplementary material for: Comorbidity Between Non-suicidal Self-Injury Disorder and Borderline Personality Disorder in Adolescents: A Graphical Network Approach
Source: Front Psychiatry. 2020 Nov 27;11:580922. doi: 10.3389/fpsyt.2020.580922 (PMC7728714; doi:10.3389/fpsyt.2020.580922)
Supplement: Supplementary file 1 [file Table_1.docx]

Supplementary Table 1. Edgewise bootstrapping table

| **node1** | **node2** | **id** | **sample** | **q2.5** | **q97.5** |
| --- | --- | --- | --- | --- | --- |
| B1 | B2 | B1--B2 | .026138 | .000000 | .161379 |
| B1 | B3 | B1--B3 | .164380 | .052248 | .196714 |
| B1 | C1a | B1--C1a | .298433 | .225951 | .390070 |
| B1 | C1b | B1--C1b | -.019447 | -.136034 | .000000 |
| B1 | C3 | B1--C3 | .095182 | .000000 | .137331 |
| B1 | E3 | B1--E3 | .041568 | .000000 | .098936 |
| B1 | leave | B1--leave | .019208 | .000000 | .072616 |
| B1 | lonely | B1--lonely | .040094 | .000000 | .120479 |
| B2 | B3 | B2--B3 | .359383 | .237099 | .471154 |
| B2 | C1b | B2--C1b | .053885 | .000000 | .226212 |
| B2 | E2 | B2--E2 | .064813 | .000000 | .209244 |
| B2 | E3 | B2--E3 | .036477 | .000000 | .171580 |
| B2 | leave | B2--leave | .030351 | .000000 | .021799 |
| B3 | C3 | B3--C3 | .040028 | .000000 | .135308 |
| B3 | E1 | B3--E1 | -.086915 | -.412029 | -.015402 |
| B3 | E4 | B3--E4 | .076818 | .000000 | .240604 |
| B3 | miss | B3--miss | .013021 | .000000 | .127069 |
| B3 | nothink | B3--nothink | .027195 | .000000 | .194521 |
| back | careless | back--careless | .073173 | .000000 | .171946 |
| back | change | back--change | .075679 | .000000 | .147432 |
| back | hurt | back--hurt | .052952 | .000000 | .100743 |
| back | leave | back--leave | .119263 | .000000 | .197025 |
| back | letdown | back--letdown | .024376 | .000000 | .109258 |
| back | lonely | back--lonely | .215100 | .218929 | .321414 |
| back | miss | back--miss | .196146 | .057625 | .248377 |
| C1a | back | C1a--back | .083485 | .000000 | .100047 |
| C1a | C1b | C1a--C1b | .346162 | .223364 | .408505 |
| C1a | C2 | C1a--C2 | .242738 | .136443 | .331375 |
| C1a | careless | C1a--careless | .011861 | -.021139 | .012257 |
| C1a | E1 | C1a--E1 | .049766 | .000000 | .168428 |
| C1a | E3 | C1a--E3 | .068607 | .000000 | .108846 |
| C1a | E4 | C1a--E4 | .021900 | .000000 | .230559 |
| C1a | leave | C1a--leave | .048804 | .000000 | .136435 |
| C1a | letdown | C1a--letdown | .056614 | .000000 | .125331 |
| C1a | lonely | C1a--lonely | .022932 | .000000 | .115701 |
| C1b | E2 | C1b--E2 | .102238 | .000000 | .259592 |
| C1b | letdown | C1b--letdown | .001336 | .000000 | .074194 |
| C1b | nothink | C1b--nothink | .035955 | .000000 | .083343 |
| C1b | strong | C1b--strong | .058299 | .000000 | .086180 |
| C2 | C3 | C2--C3 | .202107 | .100006 | .276253 |
| C2 | change | C2--change | .033803 | .000000 | .100663 |
| C2 | E2 | C2--E2 | .095243 | .000000 | .202911 |
| C2 | E3 | C2--E3 | .067647 | .000000 | .201593 |
| C2 | hurt | C2--hurt | .045968 | .000000 | .081954 |
| C2 | strong | C2--strong | .056644 | .000000 | .080282 |
| C3 | back | C3--back | .047657 | .000000 | .064554 |
| C3 | E2 | C3--E2 | .027742 | -.006118 | .094308 |
| C3 | E3 | C3--E3 | .018055 | .000000 | .132013 |
| C3 | leave | C3--leave | .024542 | .000000 | .059174 |
| C3 | lonely | C3--lonely | .157859 | .044776 | .241620 |
| C3 | miss | C3--miss | .110982 | .042576 | .173668 |
| careless | nothink | careless--nothink | .200429 | .089575 | .262570 |
| change | careless | change--careless | .033133 | .000000 | .155957 |
| change | hurt | change--hurt | .004274 | .000000 | .052805 |
| change | letdown | change--letdown | .016885 | .000000 | .046096 |
| change | lonely | change--lonely | .070546 | .022111 | .156852 |
| change | nothink | change--nothink | .018076 | .000000 | .202679 |
| days | B1 | days--B1 | .022986 | .000000 | .105947 |
| days | B3 | days--B3 | .079692 | .000000 | .208976 |
| days | C1a | days--C1a | .118719 | .000000 | .152836 |
| days | C3 | days--C3 | .386243 | .308888 | .456674 |
| E1 | E2 | E1--E2 | .139046 | .041805 | .220160 |
| E1 | E3 | E1--E3 | .104931 | .000000 | .148458 |
| E1 | E4 | E1--E4 | .149470 | .000000 | .279697 |
| E1 | miss | E1--miss | .028431 | .000000 | .035992 |
| E1 | nothink | E1--nothink | .037233 | .000000 | .148888 |
| E2 | E3 | E2--E3 | .435858 | .393819 | .580745 |
| E2 | E4 | E2--E4 | .032179 | .000000 | .221619 |
| E2 | leave | E2--leave | .067843 | .000000 | .109428 |
| E2 | nothink | E2--nothink | .108408 | .006301 | .133091 |
| E3 | E4 | E3--E4 | .212695 | .000000 | .307288 |
| E3 | nothink | E3--nothink | .010612 | .000000 | .074144 |
| E4 | leave | E4--leave | .059949 | .000000 | .205265 |
| E4 | strong | E4--strong | .068025 | .000000 | .204378 |
| hurt | letdown | hurt--letdown | .418023 | .344929 | .448292 |
| leave | change | leave--change | .108810 | .035346 | .175126 |
| leave | hurt | leave--hurt | .091850 | .000000 | .188209 |
| leave | letdown | leave--letdown | .156785 | .101004 | .224066 |
| leave | lonely | leave--lonely | .035859 | .000000 | .112290 |
| letdown | mean | letdown--mean | .020441 | .000000 | .097075 |
| letdown | nothink | letdown--nothink | .037942 | .000000 | .194077 |
| lonely | careless | lonely--careless | .091345 | .046270 | .192977 |
| lonely | hurt | lonely--hurt | .049461 | .000000 | .084557 |
| lonely | letdown | lonely--letdown | .097309 | .000000 | .188945 |
| mean | careless | mean--careless | .140572 | .058297 | .227883 |
| mean | nothink | mean--nothink | .088986 | .000000 | .126297 |
| miss | careless | miss--careless | .080724 | .027175 | .193143 |
| miss | change | miss--change | .165907 | .098442 | .292561 |
| miss | leave | miss--leave | .157407 | .065275 | .266162 |
| miss | letdown | miss--letdown | .046875 | .000000 | .108586 |
| miss | lonely | miss--lonely | .192301 | .066315 | .215018 |
| miss | nothink | miss--nothink | .045091 | .000000 | .151063 |
| strong | back | strong--back | .204728 | .155997 | .271547 |
| strong | change | strong--change | .102768 | .033868 | .194815 |
| strong | hurt | strong--hurt | .060673 | .000000 | .141491 |
| strong | letdown | strong--letdown | .018799 | .000000 | .074037 |
| strong | lonely | strong--lonely | -.008480 | -.133959 | .000000 |
| strong | mean | strong--mean | .124023 | .074939 | .232966 |
| strong | nothink | strong--nothink | .015404 | -.079065 | .082795 |

*Note. strong* = my feelings are very strong, *nothink* = I get into trouble because I do things without thinking, *miss* = I feel that there is something important missing about me, *mean* = Lots of times, my friends and I are really mean to each other, *lonely* = I feel very lonely, *letdown =* People who are close to me have let me down, *leave* = I worry that people I care about will leave and not come back, *hurt*  = I want to let some people know how much they’ve hurt me, *E4* = NSSI causes interference in other important areas of functioning, *E3 =* NSSI causes interference in academic functioning, *E2* = NSSI causes interference in academic functioning, *E1*  = NSSI causes clinically significant distress, *days* = number of NSSI days, *change*  = How I feel about myself changes a lot, *careless* = I’m careless with things that are important to me, *C3* = frequent thinking about NSSI, *C2 =* preoccupation with the act prior to self-injury, *C1b* = interpersonal conflicts prior to self-injury, *C1a*  = negative feelings or thoughts prior to self-injury, *back*  = I go back and forth between different feelings, *B3*  = to induce a positive feeling state, *B2* = to resolve an interpersonal difficulty, *B1* = to obtain relief from a negative feeling or cognitive state.
